# The role of inflammation in the pathogenesis of perinatal depression and offspring outcomes

**DOI:** 10.1016/j.bbih.2021.100390

**Published:** 2021-11-19

**Authors:** Kristi M. Sawyer

**Affiliations:** Department of Psychological Medicine, Institute of Psychiatry, Psychology & Neuroscience, King's College London, United Kingdom

**Keywords:** Perinatal, Antenatal, Postnatal, Depression, Inflammation, Breast milk

## Abstract

The role of inflammation in the pathogenesis of depression is becoming increasingly apparent, but its role in perinatal depression is less well-studied. Pregnancy and the postpartum are characterised by distinct and changing inflammatory profiles throughout, which makes the study of depression-related alterations in this period complex. This review presents literature discussing a role for the immune system in both antenatal and postnatal depression. Furthermore, literature investigating the role of the maternal immune system on breast milk composition and offspring immunological and behavioural outcomes is discussed, before concluding with suggestions for future work as this developing field grows.

## 1 Introduction

The role of the immune system in the pathogenesis of depression is becoming increasingly important (Kohler et al., 2016; Dowlati et al., 2010; Pitharouli et al., 2021; Nettis et al., 2021). The relationship between inflammation and depression appears to be bi-directional (Messay et al., 2012). As such, a high prevalence of depression is often reported in populations with medical conditions that are associated with high levels of inflammation, and as such it is suggested that inflammation may be the factor that underlies comorbidity between depression and conditions such as cancer (Sforzini et al., 2019), diabetes (Wang et al., 2019), myocardial infarction (Shang et al., 2014) and obesity (Ouakinin et al., 2018).

Less studied, however, is the role of inflammation in the pathogenesis of depression in the perinatal period. The term ‘perinatal depression’ covers both depression during pregnancy (‘antenatal depression’) and depression in the first year postpartum (‘postnatal depression’). It is possible that these two disorders, although highly comorbid and often reflecting continuation of depressive symptoms from pregnancy into postnatal life, may, at least in some cases, have distinct aetiologies, psychosocial risk factors and thus outcomes for both mother and baby (Sawyer et al., 2019).

## 2 The immune system in healthy pregnancy

The study of the role of the immune system in the aetiology of perinatal psychopathology is complicated, as profound changes occur in inflammatory functioning as part of healthy pregnancy (Leff-Gelman et al., 2016). Tight regulation of the immune system is required to prevent rejection of the “fetal allograft”, while still protecting the mother and the developing fetus from pathogens. Excessive inflammation during pregnancy is also associated with miscarriage (Christiansen et al., 2006) and other pregnancy complications, such as pre-eclampsia (Maher et al., 2019) and preterm delivery (Gilman-Sachs et al., 2018).

The three trimesters of pregnancy show different inflammatory profiles, with the first trimester being largely pro-inflammatory, the second being largely anti-inflammatory, and the final trimester reverting back to a pro-inflammatory state (Mor and Cardenas, 2010). This is in line with antenatal depression prevalence, which, according to a recent systematic review, is highest in the third trimester and lowest in the second (Okagbue et al., 2019).

Cytokines that are considered to have pro-inflammatory effects [see Table 1 for summary of cytokine actions] include tumour necrosis factor alpha (TNFα), which has been shown to be elevated in blood throughout pregnancy (compared to postpartum levels) (Kraus et al., 2010), and some cohorts show evidence of an increase as pregnancy progresses (Ross et al., 2016; Christian and Porter, 2014; Blackmore et al., 2011), but not all (Pazos et al., 2012; Ferguson et al., 2014). Whereas, interferon gamma (IFNγ; also considered pro-inflammatory) remains high in trimester 1, but then decreases as pregnancy progresses into trimesters 2 and 3 (Kraus et al., 2010, Pazos et al., 2012). Levels of interleukin-(IL-)1β show mixed trajectories, with one study reporting a linear decline across pregnancy (Ferguson et al., 2014), but another reporting that levels remain stable through the first two trimesters of pregnancy. Levels of IL-6 decline from the first to second trimester (Stokkeland et al., 2019), before increasing later in pregnancy (Ross et al., 2016; Christian and Porter, 2014; Blackmore et al., 2011; Ferguson et al., 2014). IL-8 levels show a reduction throughout the first half of pregnancy (Stokkeland et al., 2019), followed by an increase from trimester 2 to 3 (Ross et al., 2016). Finally, in an experiment where natural killer (NK) cells were isolated from patient samples and cultured (unstimulated), it was found that IL-6, TNFα and IFNγ secretion levels dropped in trimesters 2 and 3, when compared with trimester 1 levels (Kraus et al., 2012).

**Table 1:** List of common cytokines and inflammatory markers, grouped by their most common action: pro-inflammatory, anti-inflammatory or action upon cells in the adaptive immune response (Turner et al., 2014).

Pro-Inflammatory	Anti-Inflammatory	Adaptive Immunity
IL-1β	IL-6^a^	IL-2
IL-6^a^	IL-10	IL-3
IL-8	IL-12	IL-4
IL-11	IL-19	IL-5
IL-17	IL-22	IL-7
IL-18		IL-9
TNFα		IL-13
TNFβ		IL-15
IFNα		
IFNβ		
IFNγ		

Abbreviations: IL, interleukin; TNF, tumour necrosis factor; IFN, interferon.

a Note: IL-6 has been reported to have both pro- and anti-inflammatory action (Trovato et al., 2021).

Conversely, cytokines which have an anti-inflammatory effect include IL-10, which may show some reduction in levels in the first half of pregnancy (Stokkeland et al., 2019). Other biomarkers such as vascular endothelial growth factor (VEGF) and monokine chemoattractant protein (MCP-1) levels are low throughout pregnancy, in comparison to non-pregnant or postpartum levels (Kraus et al., 2010; Pazos et al., 2012).

This complex profile of cytokine expression represents the tightly regulated immune response in pregnancy, and demonstrates the importance of using measurements taken at similar gestational ages when comparing groups. It is also worth noting that the immune system may show distinct profiles in the maternal-fetal environment and systemically (Powell et al., 2017; Dimova et al., 2011).

## 3 Factors to consider in the perinatal period

There are a number of additional factors which may also alter pregnancy and postnatal levels of inflammatory biomarkers in certain groups. The most notable of such factors are briefly introduced in this section, in order that the literature presented in the remainder of this review can be interpreted while holding these factors in mind. Future studies in large cohorts should seek to investigate specific effects in these groups in more detail.

One particularly important aspect to consider is the potential difference in inflammatory profiles during pregnancy by race, when interpreting studies from different international cohorts. There is some evidence to suggest that, although inflammatory profiles are largely similar between races, the trajectories of particular inflammatory markers such as TNFα and IL-1β differ between racial groups during healthy pregnancy (Gillespie et al., 2016). Recent evidence also points to differing inflammatory responses to sleep disturbance during pregnancy between racial groups (Carroll et al., 2020), which could also be important when understanding inflammatory profiles related to perinatal depression. Taken together, these studies highlight the importance of considering racial differences in immune functioning during pregnancy, which may go some way towards explaining conflicting findings between cohorts from different countries.

In addition to this, there are various other medical and health-related factors worthy of mention. It is well-established that inflammatory functioning can also be impacted by gestation-related medical conditions such as pre-eclampsia (Maher et al., 2019), but chronic non-pregnancy conditions must also be considered. For example, given that adipose tissue is a major source of inflammatory biomarkers, and thus a bidirectional relationship exists between adiposity and depression (Shelton and Miller, 2011), inflammatory profiles may differ in obese women when compared with the general perinatal population (Christian and Porter, 2014; Steinig et al., 2017), as well as those with other conditions such as type 2 diabetes (Berbudi et al., 2020). Pharmacotherapies may also impact immune system functioning, of which selective serotonin reuptake inhibitors (SSRIs) are of particular relevance in this field, being the first-line treatment for depression and anxiety.

Finally, as will be discussed in more detail in section 4.1, psychosocial stressors and risk factors may be associated with altered inflammatory profiles, and these may not be limited to concurrent stress in the perinatal period. Indeed, experience of childhood maltreatment is reportedly associated with adult low-grade inflammation (Danese et al., 2007).

## 4 Antenatal depression

Although research is sparser for antenatal depression than non-perinatal depression, there is some evidence that antenatal depression is associated with altered levels of immune biomarkers, however not all studies find differences (Venkatesh et al., 2019; Blackmore et al., 2014). A study conducted in our group compared inflammatory marker levels between women with healthy pregnancies, and women with antenatal depression, and showed that women with antenatal depression around week 25 show higher levels of IL-6, IL-10, TNFα and VEGF (Osborne et al., 2018). Other studies have also replicated the finding that levels of IL-6 are elevated in association with antenatal depressive symptoms in the final trimester (Nazzari et al., 2020a; Osborne et al., 2019), but when looking in the first two trimesters, one study did not find an association between depressive symptoms and IL-6 levels (Blackmore et al., 2011). IL-15 and C–C Motif Chemokine Ligand 3 (CCL3) are also reportedly elevated in the final trimester in antenatally depressed women (Osborne et al., 2019).

Conversely, other studies have found lower levels of inflammatory markers in antenatally depressed women when compared with controls. Perhaps due to the exclusion of women taking antidepressant medication in our study, other work has reported contrasting findings, namely that VEGF is lower in late pregnancy in antenatally depressed women, particularly those treated with SSRIs, than in controls (Edvinsson et al., 2017). Additionally, another study reported that higher depressive symptoms in the second trimester of pregnancy were associated with lower concurrent levels of IL-1β, IL-7 and TNFα (Shelton et al., 2015).

It is important to note that the majority of studies measure inflammatory markers in the blood, as a measure of peripheral inflammation. However, in cohorts where no differences in inflammatory markers are found peripherally, it remains possible that differences in immune functioning are present centrally. In most cases measurement of the central nervous system during pregnancy is challenging, however a recent study measured cytokine levels in women about to undergo a caesarean section. In this study, although no associations were found between plasma cytokine levels and antenatal depression, higher levels of IL-1β, IL-23 and IL-33 in the cerebrospinal fluid were associated with increased odds of antenatal depression (Miller et al., 2019). This may be important, as it is conceivable that central neuroinflammation may play a more significant role in the pathogenesis of mental health disorders. However, although levels of specific inflammatory biomarkers may not be strongly correlated between plasma and cerebrospinal fluid in depressed patients, general markers of inflammation such as CRP remain correlated between the two (Felger et al., 2020), suggesting a broadly similar inflammatory profile centrally and peripherally.

Furthermore, as has been mentioned in section 3, the lack of literature consensus is likely due, at least in part, to the medical heterogeneity of the pregnant population. This was evident in a sample of obese women, where higher overall C-reactive protein (CRP) levels during pregnancy were predicted by both history of depression diagnosis prior to pregnancy and higher depressive symptoms during pregnancy. This effect was mediated by early pregnancy body mass index (Lahti-Pulkkinen et al., 2019). It is therefore possible that inflammation is a key factor underlying the possible increased prevalence of antenatal depression in this population (Steinig et al., 2017).

### 4.1 Psychosocial risk factors

Research shows that psychosocial stress is associated with inflammatory marker levels during pregnancy. One such study reported that stress was associated with increases in serum concentrations of pro-inflammatory cytokines such as IL-6 in both the first and final trimesters of pregnancy, and lower IL-10 in early pregnancy (Coussons-Read et al., 2007). Taken further, this study also showed that elevated stress and low social support were associated with increased CRP levels. It has been hypothesised that inflammation is a mechanism by which psychosocial risk factors increase the risk of depression, and that women are particularly vulnerable to these effects during the last trimester of pregnancy (a time where risk of depression is elevated) because pro-inflammatory cytokines significantly increase during the final trimester (Kendall-Tackett, 2007), however further research is required to confirm this hypothesis.

### 4.2 SSRI treatment

The current literature surrounding the impact of SSRI treatment on inflammatory functioning during pregnancy is mixed, with some studies reporting the same differences between depressed women and healthy controls, whether the depressed group are taking pharmacotherapy or not, when measured in late pregnancy (Edvinsson et al., 2017). However, this is not always the case. More in-line with findings reported in non-perinatal depression, one study has reported that women with antenatal depressive symptoms that were responsive to pharmacotherapy had lower serum TNFα levels than both those who did not respond to pharmacotherapy, and those with untreated depressive symptoms (Miller et al., 2018). In this study, where cytokines were measured between 12 and 20 weeks’ gestation, no differences were found in levels of other inflammatory markers, including IL-6, -8 and -13, IFNγ or CRP. It is unclear from these results whether pharmacotherapy itself is associated with a reduction in levels of TNFα, or if pre-existing higher levels of TNFα predispose to lack of treatment response.

## 5 Postnatal depression

Postnatal depression, that is depression within the first year after birth, is complex and heterogeneous. Postnatal depressive symptoms are often a continuation of antenatal depressive symptoms. In fact, one of the strongest predictors of postnatal depressive symptoms is antenatal depression (Faisal-Cury and Rossi Menezes, 2012; Roomruangwong et al., 2017). However, postpartum-onset depression is also prevalent, and may show a distinct aetiology, for which one hypothesis postulates a differential sensitivity to the characteristic drop in reproductive hormone levels post-birth (Bloch et al., 2000).

Indeed, many women with no previous history of depression experience ‘baby blues’, or experience depressive symptoms for the first time in the postnatal period. This makes the interpretation of the literature in this area complex, as many studies measure depressive symptoms for the first time in the postpartum, and therefore cannot make the distinction between antenatal and postnatal onset of postnatal depressive symptoms.

Postpartum-onset depression has been suggested to be, at least in some cases, a psychoneuroimmune disorder (Corwin and Pajer, 2008) and it is likely that the experience of childbirth itself has an important role to play in its pathogenesis. For example, there is some suggestion that inflammatory markers may mediate the relationship between perineal injury during childbirth and postnatal depressive symptoms (Dunn et al., 2015) Additionally, obstetric pain at various times throughout the perineal period is independently associated with depressive symptom severity at 6 weeks postpartum (Lim et al., 2020).

As has been previously stated, antenatal depression is a strong predictor of postnatal depressive symptoms (Faisal-Cury and Rossi Menezes, 2012; Roomruangwong et al., 2017). What has been less studied so far is the idea that inflammatory markers during pregnancy, which themselves are associated with antenatal depression, may be an objectively measurable predictor of postnatal depressive symptoms. One such study that sought to examine this conducted retrospective analyses and observed that TNF expression had been upregulated in both the first and third trimester of pregnancy in women who had postnatal depression (Petralia et al., 2019). IL-6 and CRP levels around the time of birth have also been reported as independent predictors of postpartum depressive symptoms (Roomruangwong et al., 2017; Liu et al., 2016). Elevated levels of regulatory T-cells in pregnancy also strongly predict postnatal depressive symptoms, with an odds ratio of 1.7 (Krause et al., 2014). It is important to note, however, that women with a lifetime history of major depression show an amplification of the characteristic post-birth increases in serum IL-6 in the early postnatal period, compared with those with no lifetime history (Maes et al., 2001), perhaps suggesting that lifetime history of depression causes an immune reactivity profile which may predispose to depression in the postnatal period.

Looking more broadly at postnatal depressive symptoms, many alterations in levels of inflammatory biomarkers have been reported. Some cytokines, such as IL-1β in urine (Corwin et al., 2008) and IL-6 and IL-8 in plasma (Achtyes et al., 2020), have been shown to be elevated in samples taken from women who reported symptoms of depression in the postnatal period. Others, such as IFNγ (Groer and Morgan, 2007), TNFα (Corwin et al., 2015; Buglione-Corbett et al., 2018) and IL-2 (Achtyes et al., 2020) have been reported to be lower in the serum of women showing symptoms of, or at increased risk of, depression in the postpartum. In many of these studies other cytokines were also measured, including those reported in other studies to show differences, but with no differences found, once again reflecting, as during pregnancy, the heterogeneity of the population and findings.

## 6 Offspring outcomes

### 6.1 Infant and child outcomes

The particular importance of perinatal mental health is that any biological alterations occurring because of mental health problems during this period not only impact the mother herself but may lead to changes in the developing fetus and subsequent offspring. There are examples in the literature of maternal depression being associated with offspring immune functioning. For example, in a large cohort study, antenatal depression, but not postnatal depression, was associated with reduced infant faecal secretory immunoglobulin A (sIgA) concentrations, independently of breastfeeding status (Kang et al., 2020). These alterations as a result of maternal affect may be especially salient in certain subgroups, for example in a study which compared preterm with term births, it was found that in the preterm group only, maternal negative emotions (defined as depressive symptoms or negative affect) were positively associated with levels of IL-6, -8, -10, −13 and −18 in the cord blood, as well as maternal levels of IL-6 and -8 (Fransson et al., 2012). Interestingly, a recent study reported that expression of cytokine genes in specific neonatal brain regions modulated the association found between maternal antenatal depression and neonatal brain volume, for example in the right hippocampus (Wu et al., 2020).

Additionally, some studies report that differences in maternal levels of inflammatory biomarkers, which, as has already been discussed, are associated with maternal depression, may impact offspring growth and development. Indeed, one study reported higher maternal IL-6 levels were associated with smaller head circumference at birth (Nazzari et al., 2019). Some studies report that head circumference may be a proxy for brain development (Cooke et al., 1977), however in the aforementioned study no association was found between reduced head circumference at birth and infant behavioural regulation. Finally, maternal pro-inflammatory cytokines in the third trimester (IL-6, TNFα, MCP-1) mediate the association between higher maternal antenatal depressive symptoms and maternal report of infant negative affect, even when controlling for maternal affect during the postnatal period (Gustafsson et al., 2018).

Looking further into child development, the impact of immune system correlates of perinatal depression may have longer-term impacts on the offspring which last beyond infancy. In fact, maternal depression has been associated with child sIgA levels at age 10, and such levels were also positively correlated with child internalising and externalising symptoms at 10 years (Ulmer-Yaniv et al., 2018).

Evidence from rodent models provides more weight to the notion that maternal inflammation in pregnancy is associated with offspring outcomes. Maternal infection during pregnancy is associated with increased depressive- and anxiety-like behaviours in male offspring (Enayati et al., 2012), and in response to late-gestation immune activation, changes can be observed in neuroimmune gene expression immediately postpartum, in females (Posillico and Schwarz, 2016). This idea has also been recently supported by human cohort studies, including a study in a French birth cohort, which showed that higher levels of IL-7 in the cord serum at birth were associated with a lower likelihood of child anxiety and depression symptoms between age 3 and 8 years (Galera et al., 2021).

Conversely, while maternal inflammation is associated with offspring behaviour, maternal behaviour has also been shown to be related to offspring immune functioning. Exposure to a rodent maternal care deprivation paradigm was associated with increased levels of IL-1β, IL-6 and TNFα in the brain and serum of offspring, and reduced levels of IL-10, suggesting both central and peripheral inflammation (Réus et al., 2017).

### 6.2 Breastfeeding

Breast milk plays an important role in the development of the offspring immune system. *In utero*, a mother transmits antibodies to the developing fetus via the placenta, but after birth, breast milk is the only route through which antibodies can be transmitted from mother to baby (Di Benedetto et al., 2020). Importantly, research has been conducted to investigate the impact of maternal mental health on milk composition, and relevant to this review, the content of immune-related factors in the breast milk. One of the most well-studied molecules in this regard is sIgA, but results are mixed in terms of the direction of the effect that postnatal depressive symptoms have on its concentration in the breast milk: one study showed no association (Groër et al., 1994), one showed increased levels in association with higher depressive symptoms (Hart et al., 2004), and one showing lower levels (Kawano and Emori, 2015). The latter study is in line with a study measuring sIgA in infant faeces, which found that infants born to mothers who had depressive symptoms at any time in the perinatal period had lower faecal sIgA concentrations. This association was not present independently of breastfeeding (Kang et al., 2018). Although more research needs to be conducted in this area to understand these conflicting findings, the composition of breast milk could be important, as increased sIgA levels in the breast milk have also been associated with better performance on neonatal orientation measures (Hart et al., 2004).

In addition to antibodies, the breast milk also contains other immune-related factors, however there seems to be little evidence so far for an association between postnatal psychosocial distress and levels of immune factors (interleukins, interferons, growth factors) in breast milk (Aparicio et al., 2020), although this study also reported no difference in immunoglobulins.

## 7 Future directions

Although a role for the immune system has been well-established in the pathogenesis of depression, further research needs to be conducted to investigate how the immune system is relevant in perinatal depression. As is the case for non-perinatal depression, it is likely that there will be individual differences in the degree to which the immune system plays a role in perinatal depression pathogenesis (Raison et al., 2006). Large cohorts should also seek to investigate the role of the immune system in differences shown between suggested perinatal depression subtypes (Putnam et al., 2017) and symptom trajectories (Wikman et al., 2020), both of which may have distinct risk factors. Taken further, future studies should seek to understand whether common changes are present across different perinatal mental disorders, including more severe disorders such as postpartum psychosis, which may share some biological similarities with depression, including a role for the hypothalamic-pituitary-adrenal axis (Hazelgrove et al., 2021). Pregnancy and birth evoke a multitude of immune-related changes in order to progress safely for both mother and baby, therefore the differing inflammatory profiles observed at different stages of pregnancy and the postnatal period make the interpretation and synthesis of findings concerning depression-related alterations to the immune system complex. This review has brought together findings involving many different immune-related molecules at different stages of the perinatal period, and many studies have reported conflicting findings. As such, given the differing inflammatory profiles between pregnancy trimesters, it is possible that conflicting results are due, at least in part, to differing collection timepoints. Thus, future studies should seek to collect inflammatory biomarker data at multiple points throughout pregnancy in order to achieve a more holistic overview of pregnancy trajectory. Furthermore, as has been previously suggested, an important aspect of future research in this field will include investigation into which inflammatory profiles or shifts are of most relevance in the pathogenesis of perinatal depression (Osborne and Monk, 2013) and seek to consolidate or explain conflicting findings.

Further work is also necessary to understand the interplay of the immune system with other major biological systems in the aetiology, prognosis and treatment of perinatal depression. These include the hypothalamic-pituitary-adrenal axis, of which cortisol is the main biomarker. Cortisol is considered to be an immunosuppressing hormone, therefore it will be important to understand more about why studies in perinatal depression report concurrently elevated levels of cortisol and cytokines (Osborne et al., 2018). Recent research has also begun to include sex hormones as important factors which may be inter-related with immune system molecules (Sha et al., 2021), including research showing a relationship between lower antenatal levels of allopregnanolone (a progesterone metabolite) and postnatal depression (Osborne et al., 2017), but further research is warranted in this area. As in non-perinatal depression, the relationship between elevated cytokine levels and perinatal depression has also been suggested to be moderated by a number of metabolic pathways such as the tryptophan pathway (Nazzari et al., 2020b), therefore future studies should seek to investigate these relationships more thoroughly.

Finally, other areas of research in the field of perinatal mental health and offspring outcomes have highlighted the importance of offspring sex, as some exposures have been shown to have sexually dimorphic effects on offspring outcomes. More needs to be done to understand the impact of offspring sex on maternal inflammatory processes, whether offspring sex plays a role in the programming of the fetal immune system and the effect of maternal immune functioning during pregnancy and breastfeeding on offspring behavioural outcomes.

Nevertheless, there is increasing evidence that the immune system plays an important role in the pathogenesis of perinatal depression, and therefore immune biomarkers could represent exciting novel therapeutic targets for depression in the perinatal period (Achtyes et al., 2020).

## Statement of funding

KMS′ work has been supported by the following grants: the 10.13039/501100000272UK National Institute for Health Research (NIHR) Biomedical Research Centre at the South London and Maudsley 10.13039/100009362NHS Foundation Trust and King's College London; The Lullaby Trust (formerly known as the Foundation for the Study of Infant Deaths); and the 10.13039/100016210Psychiatry Research Trust. KMS was funded by an 10.13039/501100000265MRC Doctoral Training Programme Studentship and is now working on a 10.13039/100010269Wellcome Trust funded project.

## Declaration of competing interest

KMS declares no conflicting interests.
